# Age Differences in Response to Framed Side Effects Information About Hypothetical Medications

**DOI:** 10.1093/geroni/igaa057.1622

**Published:** 2020-12-16

**Authors:** Alyssa Minton, Madeline Nievera, Nathaniel Young, Joseph Mikels

**Affiliations:** DePaul University, Chicago, Illinois, United States

## Abstract

Framing equivalent information as a gain (e.g., 90% survival rate) or a loss (e.g., 10% mortality rate) can differentially impact judgments and decision making, such that people make more favorable judgements when information is presented as a positive gain versus a negative loss. The current study investigated how framing and age influences evaluative judgments of hypothetical medications used to treat common health issues when the equivalent probability of experiencing a particular side effect was presented as a gain (e.g., “86% of people who took this medication did not experience rash outbreaks”) or a loss (e.g., “14% of people who took this medication did experience rash outbreaks”). Younger and older adults were presented with health pamphlets for hypothetical medications with three unique side effects for each and indicated the medication’s perceived riskiness, how positively and negatively they felt about the medication, and their likelihood to take the medication. Numeracy, risk-taking behavior, and current affective state were also measured. When information was presented in a loss frame, people reported more negative feelings about the medication, leading to greater perceived riskiness, IE = -.785, SE = .13, p < .001. Age indirectly influenced likelihood via the positive feelings about the medication, IE = .349, SE = .14, p = .013. Younger adults felt more positively about the medications than older adults, leading to an increased willingness to take the medication. These findings provide insight into how framing and age can differentially influence evaluative judgments, perceived risk, and willingness to take medication.

